# A Panorama of Cloud Platforms for IoT Applications Across Industries

**DOI:** 10.3390/s20092701

**Published:** 2020-05-09

**Authors:** Sami Yangui

**Affiliations:** LAAS-CNRS, Université de Toulouse, INSA, 31400 Toulouse, France; yangui@laas.fr

**Keywords:** cloud computing, fog computing, Internet of Things (IoT), Industrial IoT (IIoT), Platform as-a-Service (PaaS)

## Abstract

Internet of Things (IoT) applications can play a critical role in business and industry. Industrial IoT (IIoT) refers to the use of IoT technologies in manufacturing. Enabling IIoT applications in cloud environments requires the design of appropriate IIoT Platform as-a-Service (IIoT PaaS) to support and ease their provisioning (i.e., development, deployment and management). This paper critically reviews the IIoT PaaS architectures proposed so far in the relevant literature. It only surveys the architectures that are suitable for IIoT applications provisioning and it excludes regular IoT solutions from its scope. The evaluation is based on a set of well-defined architectural requirements. It also introduces and discusses the future challenges and the research directions. The critical review discusses the PaaS solutions that focus on the whole spectrum of IoT verticals and also the ones dealing with specific IoT verticals. Existing limitations are identified and hints are provided on how to tackle them. As critical research directions, the mechanisms that enable the secure provisioning, and IIoT PaaS interaction with virtualized IoT Infrastructure as-a-Service (IaaS) and fog computing layer are discussed.

## 1. Introduction

Internet of Things (IoT) refers to the ever-growing pervasive network of various objects—such as Radio-Frequency IDentification (RFID) tags, sensors, actuators, mobile phones, etc.—connected over the Internet [1]. These objects are able to interact with each other and cooperate with their neighbors through unique addressing schemes seeking to reach a common goal. The Industrial Internet of Things (IIoT), also known as the Industrial Internet, is the use of Internet of Things (IoT) technologies in manufacturing [2]. The main motivation behind the IIoT is that autonomous and smart machines are more efficient than humans when it comes to the accurate capture, analysis and operation of data. From the companies side, IIoT enables the saving of time and money, supporting business intelligence efforts and understanding and reacting to the novel potential markets. From the end-user side, this emerging infrastructure of objects enable a plethora of novel applications. Autonomous transportation, robotics, supply chain traceability and healthcare are among the several examples. Obviously, efficiency in resources usage, scalability, elasticity and easy development are among the key requirements for such applications. Notably, cloud computing might aid in meeting these requirements. Cloud computing is an emerging model for enabling ubiquitous, convenient and on-demand network access to a shared pool of configurable computing resources. These resources should be swiftly provisioned and released with minimal management effort [3]. Cloud computing handles and delivers resources according to three predefined service models: (a) Infrastructure as-a-Service (IaaS), (b) Platform as-a-Service (PaaS) and (c) Software as-a-Service (SaaS).

Generally speaking, end-user applications, including IIoT applications, are provisioned in cloud settings using PaaS and offered as SaaS. The provisioning process consists of three phases: (a) Developing the end-user application sources; (b) deploying them on PaaS resources; and (c) managing (including executing) the applications to optimize its performance and the resources usage [4]. Specifically, the PaaS provides the services that offer the resources needed to provision consumer-created or acquired applications implemented using programming languages, libraries and tools supported by its provider [3]. These resources are deployed in the underlying IaaS. The interaction between the PaaS and IaaS is enabled through well-defined interfaces. Specifically, a PaaS abstracts the IaaS resources and offer a re-usable framework, which provides application platform components as-a-Service. Examples of components are containers and DataBase Management Systems (DBMS). Containers are the service engines necessary for applications hosting and execution. DBMS handle and manage persistent data. An example of PaaS is Cloud Foundry(www.cloudfoundry.org). With Cloud Foundry, the applications could be implemented, for instance, with Java programming language, Java Spring library and services integration tools. The interactions between Cloud Foundry and IaaS could be done via Amazon Web Services (AWS).

This manuscript is devoted to the PaaS for IIoT applications provisioning. It identifies evaluation criteria and rely on them to review and evaluate the proposed IIoT PaaS architectures in the relevant literature. It also discusses the research directions. The reader should note that this manuscript exclusively surveys IIoT PaaS architectures and excludes the regular IoT PaaS from its scope. Basically, IoT and IIoT have separate areas of interest. On one side, IIoT connects critical machines and implements systems in which failure often results in life-threatening or other emergency situations. On the other side, IoT connects consumer-level devices with low risk impact in case of failure and where breakdowns do not immediately create risky situations. In addition to that, IIoT is developed with the concept of using sophisticated and precise devices to extend existing manufacturing while IoT relies on cheap and simple smart devices to mainly improve consumer convenience. IIoT applications are intended to handle critical machines, precise devices with advanced controls and analytics capabilities.

These fundamental differences make the IoT PaaS architectures and solutions not suitable for IIoT applications provisioning. This explains why emerging IoT PaaS solutions (e.g., Microsoft Azure IoT Suite, Google IoT Platform, IBM Watson IoT Platform, AWS IoT Platform and Cisco IoT Cloud Connect) are seldom used to host and execute IIoT applications. The suitable architectures for IIoT PaaS are still in their early development stages. In the literature, there are several papers that surveys the IoT cloud platforms (e.g., see [5,6,7,8,9]). This manuscript exclusively surveys the literature on PaaS architectures for industrial Internet. It relies on a set of IIoT use cases and PaaS reference architecture to derive the evaluation criteria. The existing IIoT PaaS are reviewed and evaluated in the light of these criteria.

The next section introduces IIoT use cases and derives architectural requirements for IIoT PaaS with respect to the PaaS reference architecture. Section 3 reviews the works that propose PaaS supporting the whole spectrum of IoT verticals. Section 4 reviews the works that propose PaaS for specific IoT verticals (e.g., PaaS for robots and PaaS for Wireless Sensors Network). Section 5 is devoted to the synthesis and the research directions. Section 6 concludes the paper and presents the planned future work.

## 2. Use Cases, Design Considerations and Evaluation Requirements

This section first introduces a set of illustrative IIoT use cases while emphasizing the perspective of their provisioning in cloud setting. This is followed by the discussion of the associated design specifications that need to be taken into consideration. Finally, it identifies and derives the architectural requirements to meet to provision IIoT applications over cloud platforms. These requirements were used to evaluate the reviewed papers.

### 2.1. Use Cases

IIoT use cases range from connected vehicles to healthcare, fleet management and smart metering [10]. For illustration purposes, three specific use cases are described in detail in this subsection.

The first use case describes an autonomous car that is riding in a smart city [11]. The car is equipped with a set of IoT devices (e.g., cameras, light detection and ranging sensors) and actuators (e.g., throttle and brakes). According to the specification of the fifth generation of wireless communications technologies, mobile entities such as autonomous cars exchange services and data with edge and cloud providers during operation. In addition, autonomous cars may interact with other entities in the city (e.g., other cars, smart traffic lights and parking sensors) for efficiency, cost-effectiveness and/or security purposes. For instance, emergency vehicles could send an alert signal to a smart traffic light to get the priority when arriving to a road intersection.

The second use case describes a robotic prescription dispensing and medication delivery system [12]. It relies on patients information and data collected from Body Area Network (BAN) to automatically select the appropriate medication and dosage to be prescribed to patients. Data storage and parsing, as well as computation occur in the cloud. Once the medications are selected, delivery robots are dispatched to get the medication and deliver them to the patients. The robots control actions are calculated and sent from the cloud.

The third use case describes a predictive maintenance system for planes. This system aims to provide automated, secure and rapid maintenance for planes. It enables leveraging streaming data from sensors and devices in the planes to assess current conditions, recognize warning signs and deliver alerts. Specifically, the collected data are sent to analytics services in the cloud that parses the data and relies on machine learning and trained algorithms to detect the upcoming failures for the planes components. These services interact with other modules that are responsible of automatically creating maintenance timelines before problems occur.

### 2.2. Design Considerations

**Figure 1:**
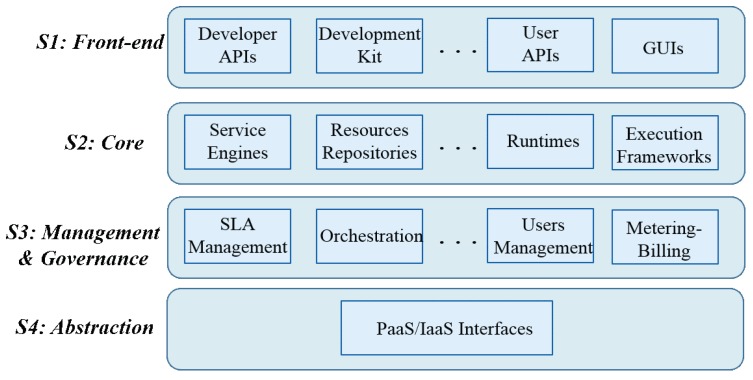
IBM Platform as-a-Service (PaaS) reference architecture.

IIoT applications are considered as part of Cyber Physical Systems (CPSs) [13]. CPSs involve the combination of several systems that aim to control physical processes. These systems are auto-adaptive in real-time and are capable to adjust and reconfigure itself based on the operation feedback. Basically, this is what it makes the provisioning of IIoT applications in the cloud very challenging. Indeed, CPSs are not necessarily connected to the Internet and thus, not necessarily connected to the cloud platforms that are deployed on or to the IoT devices that they interact with. Provisioning IIoT applications in cloud platforms while meeting the CPSs specifications need the satisfaction of several architectural requirements. Basically, existing PaaS architectures need to be extended and/or adapted to support IoT applications and address these specific characteristics. The starting point naturally would be a PaaS reference architecture. In this work, the IBM PaaS reference architecture [14], that was inspired from the Cloud Computing Reference Architecture (CCRA) [15], is considered as starting point. An overview of this architecture is depicted in Figure 1. It consists of four strata: *Front-end*, *Core*, *Management and Governance* and *Abstraction Interface*.

The *Front-end* stratum exposes northbound APIs for developers and end-users. The *Core* stratum involves the several runtime environments (e.g., containers) supported by PaaS and is necessary for applications hosting and execution. The *Management and Governance* stratum implements the PaaS business support system and involves the entities that handle PaaS and applications management (e.g., monitoring and scalability). Finally, the *Abstraction* stratum exposes southbound APIs, enabling the PaaS to interact with the underlying IaaS to provision the required compute, storage and networking services. IaaS is the actual dynamic pool of the virtualized resources used by the applications.

### 2.3. Requirements

For the specific case of IIoT applications, the PaaS should support additional features and capabilities so that it supports the specificities of IoT applications, as well as the characteristics of CPSs. The IIoT PaaS should make the bridge and establish the missing connection between the IoT applications running over it and the CPSs. The target extensions need to be added at all levels of the PaaS reference architecture. To that end, the needed requirements are identified and discussed for each stratum of the PaaS reference architecture.

At the *Front-end* stratum, appropriate APIs and development kits are required to support the provisioning of the wide range of IIoT applications (**R1**) such as the various use cases presented in Section 2.1. This could be done based on the concept of IoT verticals. In classical environments, IoT applications are provisioned as physically isolated systems while these applications are provisioned as logical verticals in the cloud [16,17]. The motivation behind that is to enable efficient resources usage and scalability when managing the applications. An IoT vertical is a logical entity that involves an IoT application with its underlying devices and, eventually, the required gateways in some cases.

At the *Core* stratum, the IoT PaaS requires additional mechanisms and tools to enable interactions with a fog layer (**R2**). Fog computing is a novel concept that addresses the excessive delays when operating latency-sensitive applications in the cloud such as IoT applications [18]. It provides additional computation and storage resources closer to the end-users and/or IoT devices to reduce the delays. IoT PaaS should be able to provision IoT application components either in the cloud or the fog. For instance, considering the autonomous car use case, part of the collected data from the car neighborhood could be sent to the closest fog devices (e.g., road-side units) for fast processing such as obstacles detection while the rest of the data could be sent to the remote cloud that is responsible of processing the compute-intensive tasks such as the itinerary calculation.

At the *Management and Governance* stratum, the IoT PaaS should involve additional features and business support that would enable it to: (i) federate and cooperate with other IoT cloud providers (**R3**) and (ii) ensure control and security during operation (**R4**). As for (**R3**), a proper IIoT PaaS should provide and support inter-cloud communication so that it can use/offer services and data to other providers. The idea have already been shown to be effective in cases like the global Internet and wireless telco networks. Specifically, federated IIoT PaaS solutions will enable an industrial “cloud of clouds” perspective where any single common functionality would combine many different individual clouds into one seamless mass in terms of on-demand operations. Based on [19], this could be enabled through open interfaces that would govern the resources portability, metering and billing from one cloud provider to another (e.g., see RESERVOIR [20] for IaaS resources portability, see COAPS [21] for PaaS resources portability). In addition, this federative approach is key to achieve one of the most important industry 4.0 vision, i.e., a universal decentralized computing environment [22]. Inter IIoT cloud communications make up a ubiquitous, multi-provider infrastructure (mesh of service providers) that are interconnected to deliver decentralized computing and storage capabilities where everything is driven by constraints and agreements. This is mandatory to match the complex and sophisticated business needs of IIoT and optimize enterprise IT service delivery. For instance, considering the autonomous car use case, the running car services should be able to interact with any relevant IoT applications provisioned over the city resources, independently of their hosting provider, to get useful data during a given trip (e.g., accidents and recommended parking facility). When it comes to (**R4**), ensuring control and security during operations is critical to achieve proper procedures for industrial environments where agility, cooperation between several entities, efficient control and security are needed [23]. Provisioning IIoT applications in such dynamic and distributed system requires tackling challenges and threats raised by the management of the digital identity across diverse organizations, security domains and application platforms [24]. For instance, considering the healthcare use case, the IoT PaaS should support appropriate mechanisms that ensure the security and the privacy of the vital data of patients.

At the *Abstraction* stratum, the IoT PaaS should be able to: (i) support the heterogeneity of the IoT devices and to cover their whole spectrum (**R5**) and (ii) it should be able to interact with novel IoT infrastructures when provisioning the IoT applications (**R6**). IoT IaaS is fundamentally different from the traditional IaaS due the significant differences between classical virtual machines, the classical IaaS rely on, and the virtual IoT. For instance, in the specific case of Wireless Sensors Network (WSN) and Virtual Sensor (VS), VMs aim at sharing the physical machine resources (e.g., computing, storage), whereas VSs use the host sensor resources with the aim of sharing its sensing capabilities (e.g., temperature, light and humidity) [25]. As an example, IoT PaaS should be able to provision several maintenance applications that might use the capabilities of different VSs running on top of the same physical sensors in the plane. For instance, two different applications (i.e., engine temperature control and combustion chamber pressure control could use the same physical sensor but rely on two different VSs, with each VS configured based on the specific needs of the application (e.g., temperature unit and periodicity) to monitor the temperature and flow rate of exhaust fan discharge.

The reader should note that, according to CCRA and, in addition to meeting these requirements, the PaaS architecture for IIoT applications should also ensure proper vertical interactions between all the strata. For instance, the control and security capabilities models of the *Management and Governance* stratum should be implemented at the *Core* stratum and exposed to prospective end users through appropriate APIs at the *Front-end* stratum. This way of design is quite intuitive and is inline with the early introduced IoT verticals concept. In the rest of this paper, an evaluation of the relevant literature is done in the light of the defined requirements. The critical review discusses the architectures that focus on both (i) the whole spectrum of IoT verticals and (ii) the ones dealing with specific IoT verticals.

## 3. PaaS for Whole Spectrum of IoT Verticals

**Figure 2:**
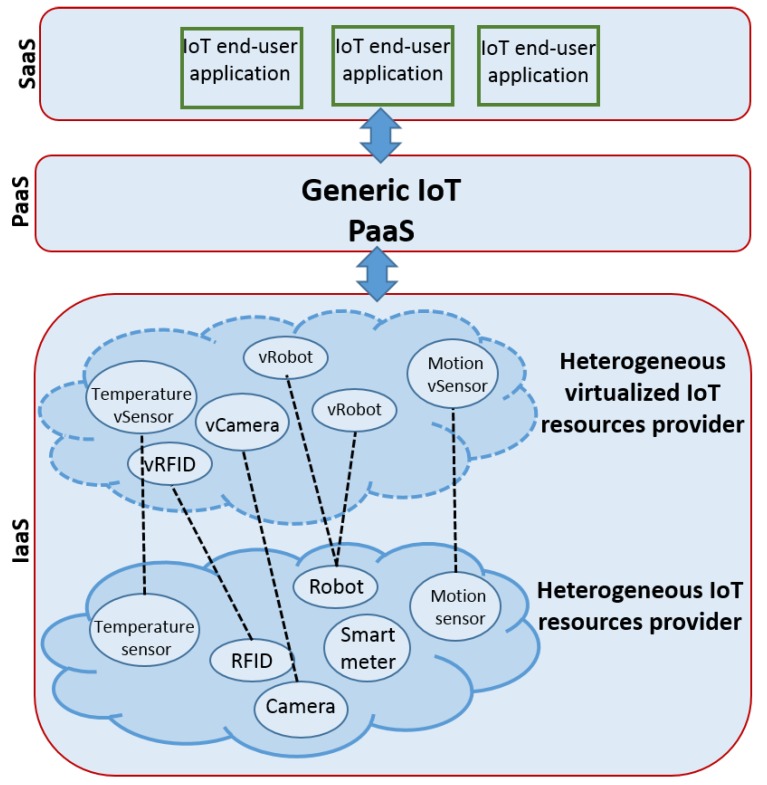
Generic PaaS for whole spectrum of the Internet of Things (IoT) verticals.

The PaaS for the whole spectrum of IoT verticals are able to host and run end-user applications that interact and use any virtualized IoT resources as shown in Figure 2. The heterogeneity of the objects is supported by the IoT PaaS itself. The related work could be classified into three groups of approaches: (i) The virtual vertical-based approach; (ii) the services compositions approach; and (iii) a set of work in which contributors focus only on a sub-set of functionality. In what follows, the works related to each of these groups are discussed and evaluated.

### 3.1. Virtual Vertical-Based Approach

In [17], the authors propose a PaaS architecture and implementation based on the concepts of virtual verticals. A virtual vertical consists of a set of resources and services, provided by a given IoT IaaS in the form of an isolated virtual solution to the PaaS. The vertical solution can be assimilated to the hosting environment of IoT end-user applications and can be customized in relation to the physical objects these applications use. The technical PaaS services (e.g., monitoring) can be shared horizontally between the vertical solutions. Specifically, the proposed PaaS offers vertical solutions for IoT end-user applications hosting and resources management. Each vertical solution is domain-specific. It is therefore possible to take into account the differences and the specificities of the objects when controlling the applications. According to the authors, the control of the application is the process of activating and managing the PaaS resources it uses, such as security and scalability management, and the data it generates in the context of the virtual solution that it belongs to. End-user applications control is ensured by the “application context management” module. This module interacts with the objects through “gateways” and “mediators”. The “gateways” allow for providing the drivers and protocol stacks for the communications with the objects. The “mediators” domain mediates the interfaces between different gateways in the same application domain to cover the diversity of all domain-specific data models.

For the end-user application deployment (including driver installation and gateway configuration), the same authors propose in [16] to extend Topology and Orchestration Specification for Cloud Applications (TOSCA) specifications in order to support IoT end-user applications deployment and management in the same PaaS. TOSCA provides specifications to describe applications and their hosting environment topologies as typed graphs. These specifications simplify the management of applications and their related components reusability. In [16], the authors use TOSCA to formally describe the IoT end-user applications topology and its dependencies in terms of required resources and configuration actions. This allows them to select the most appropriate vertical solution to deploy an end-user application. In addition, it allows the automation of the applications deployment and management instead of the current manual handling in classical environments. It allows to avoid the current tedious configuration and management tasks performed in a case-by-case manner, due to the underlying heterogeneity of IoT infrastructures. Furthermore, the use of TOSCA model could automate the interoperability with other platforms assuming they support TOSCA.

This work meets the first requirement (R1) concerning the support of wide range of IoT end-user applications. The proposed TOSCA extension enables describing any end-user IoT application topology in terms of interacting processes. It also allows the description of their management processes using the TOSCA plans. This work does not meet the requirement concerning the fog layer support (R2), although the “event processing” module enables real-time events analysis. In fact, this module considers only the availability and reliability of the used objects. Moreover, the collected data are intended for the PaaS solution rather than for the end-user applications (e.g., to restore the connection with a disconnected sensor). This work partially meets the third requirement concerning the federation with other IoT clouds (R3). Indeed, the use of TOSCA enables the design of interoperable IoT applications and resources between heterogeneous cloud providers. However, this assumes that the involved providers support TOSCA, which is not really common with the existing cloud providers in the market. This work meets the requirement concerning the control and security support (R4). Security model and strategy can be integrated with this solution as part of the technical PaaS services if it is needed in a specific IoT vertical. This work meets the requirement concerning the support of the whole spectrum of IoT devices (R5) thanks to the use of “mediators” and “context management” modules. Finally, this work does not meet the requirement concerning the support of virtualized IoT services (R6). Although, the virtual vertical solutions notion is favorable to the use of virtualized IoT resources, the authors do not highlight this perspective in this work. Even the introduced use cases (i.e., temperature control and presence-based light control) are based on physical devices.

In [26], the authors propose a system architecture that models a vertical IoT silo for environmental and health monitoring. It relies on wearable sensor network system to monitor the safety and health risks for workers in construction environments. The proposed architecture consists of three layers. The layer in the southbound involves the IoT wearable sensor nodes. The layer in the northbound represents the remote cloud system at the back-end. It includes data storage servers and further functionalities, such as Web monitoring and mobile applications. The layer in between implements the required gateway for the communication and the data routing from the IoT devices to the cloud applications. It also acts like a local fog server, responsible of pre-processing sensor signals and raising early alerts in case of emergency.

This work does not meet the first requirement (R1) concerning the support of wide range of IoT end-user applications. It is designed to only provision the safety and health monitoring applications. This work partially meets the requirement concerning the fog layer support (R2). The gateway in the middle architectural layer hosts and executes the application components responsible of the data pre-processing and the alerts management. However, it is not possible to include additional fog nodes in this layer. This work does not meet the third requirement concerning the federation with other IoT clouds (R3). It is designed as a closed IoT silo and cannot interact with other IoT clouds. This work meets the requirement concerning the control and security support (R4). It includes privacy and security mechanisms for the data transmitted from the IoT devices to the application components in the fog and the cloud. This work does not meet the requirement concerning the support of the whole spectrum of IoT devices (R5). It only supports the four environmental sensors, such as the CO${}_{2}$ sensor and the UV sensor, that are integrated to this architecture. Finally, this work does not meet the requirement concerning the support of virtualized IoT services (R6). All the used IoT and sensors devices are physical.

### 3.2. Services Compositions Approach

In [27], the authors propose an open source PaaS for smart city end-user applications. The PaaS is designed as part of the COMPOSE EU FP7 project(https://compose-project.eu/) and provides features to discover and compose tools and services in order to build and deploy end-user applications upstream smart objects. The architecture of the COMPOSE PaaS consists of a marketplace, a runtime engine and an ingestion layer. The marketplace provides graphical user interfaces for developers to operate the reusable services. It allows to develop applications through IoT workflows and libraries by specifying the appropriate services discoverable from the PaaS. The services that are not supported by the PaaS can be provided and published by the developers. The runtime engine is the component that is responsible for deploying the end-user applications. This engine is based on an extension of Cloud Foundry PaaS. It instantiates the required services described in the workflow and deploys them over the PaaS resources before performing necessary compositions in order to configure and set the interactions between the selected services. The compositions and the configuration operations are based on information in the service object description module and the management component in order to consider the specificities of the objects referenced by the instantiated services. The ingestion layer provides the APIs and the interfaces to connect and make the services interact with the smart objects. It implements several communication models and protocols in order to cover the many various objects in the COMPOSE infrastructure (e.g., actuators and sensors).

COMPOSE PaaS meets the requirement concerning the support of heterogeneous IoT applications(R1). Any IoT application could be modeled with elementary services that are composed together to implement the end-to-end target application. This work does not meet the requirement concerning the interaction with fog layer (R2). Similarly, it does not meet the requirement concerning the federation with other IoT clouds (R3). Indeed, COMPOSE PaaS is strongly coupled and designed to only communicate with COMPOSE infrastructure. This work meets the requirement concerning the control and security support (R4). Security modules, when required, can be provided by developers and published by COMPOSE through its marketplace to be used with IoT applications. This work meets the requirement concerning the support of the whole spectrum of IoT devices (R5). The heterogeneity of the IoT devices is hidden thanks to the use of the service object description module. Finally, this work does not meet the requirement concerning the support of virtualized IoT services (R6). The marketplace services are only related to physical smart objects.

In [28], the authors propose an architecture that virtualizes IoT devices and bind them with IIoT applications as a service. The considered applications are from the industrial Internet, i.e., anti-fire and Heating, Ventilation and Air Conditioning (HVAC) applications. The proposed architecture is layer-based. The physical layer involves the heterogeneous physical IoT devices. The virtual IoT layer hosts the virtualized IoT services. These services are published in repository acces engine in the IoT management layer. The running IoT applications discover and select the required IoT services from that repository. Depends on the applications needs, the selected services might be elementary or composed of several elementary services. The composition is handled by the orchestrator with respect to the orchestration plans published with the virtualized IoT services.

This work meets the requirement concerning the support of heterogeneous IoT applications (R1). This work does not meet the requirement concerning the interaction with fog layer (R2). Only IaaS/PaaS interfaces are designed in this architecture. Moreover, it does not meet the requirement concerning the federation with other IoT clouds (R3). The architecture is designed as a set of closed IoT vertical that cannot be extended and/or merged with external entities that belong to other clouds. This work does not meet the requirement concerning the control and security support (R4). This work meets the requirement concerning the support of the whole spectrum of IoT devices (R5). The authors prove this capability in the developed prototype by integrated two different types of IoT devices (i.e., sensors and drones) and bind them with different IoT applications. Finally, this work meets the requirement concerning the support of virtualized IoT services (R6). All the the considered IoT services in this work are virtualized from physical devices and bound to the applications as a service.

In [29], the authors present Smart Water Management Platform (SWAMP) for precision irrigation in smart agriculture. The designed architectural mainly focus on replicability and scalability. The SWAMP platform uses FIWARE(https://www.fiware.org/) components and relies on semantics to compose and configure customizable services that specialize generic analytic services into particular techniques for different types of irrigation and water distribution management. The goal of services compositions is to bring flexibility and adapt the operated applications to specific countries, soils, climate and so on. The SWAMP architecture is layer-based. It consists of five layers. The first layer contains the IoT devices. The second layer implements the security and the communication protocols/channels. The third layer takes care of the storage services, data analytics and machine learning. The fourth layer contains the generic and customizable services, as well as the water irrigation distribution models. Finally, the fifth layer delivers the concrete and well-configured applications.

This work meets the requirement concerning the support of heterogeneous IoT applications (R1). The concept of generic and customizable services models enables the provisioning of various IoT applications depending on the end users needs. The paper details the irrigation and water distribution management use case. However, the same principle can be applied to any kind of other IIoT applications. This work partially meets the requirement concerning the interaction with fog layer (R2). It supports the provisioning of some services over fog nodes at the third layer of the architecture. However, it only supports the integrated IoT devices (SWAMP fog hub) as fog nodes. This work does not meet the requirement concerning the federation with other IoT clouds (R3). It only supports provisioning services over the SWAMP cloud. This latter is able to communicate with different external data sources for knowledge enrichment such as meteorological data and agriculture yield historical data. This work meets the requirement concerning the control and security support (R4). It uses FIWARE security components at the second layer to ensure data security. This work meets the requirement concerning the support of the whole spectrum of IoT devices (R5). However, it does not meet the requirement concerning the support of virtualized IoT services (R6). All the used IoT devices are physical.

### 3.3. Focus on Sub-Set of Functionality

Some papers focused on a single aspect of provisioning IoT end-user applications in PaaS. They only treat single aspects of IoT applications provisioning in PaaS (e.g., data management in [30], power management in [31] and cost-efficient storage [32]).

In [30], the authors introduce an IoT-oriented data storage framework at the PaaS level. The proposed framework allows (i) processing the high throughput of the data sent by the objects to the running end-user applications and (ii) horizontally scaling the data management resources in order to support the huge volume of the data. The proposed data storage framework handles heterogeneous data collected from various objects (e.g., RFID readers and ubiquitous sensors). These data are different in terms of structures, schemes, accessing modes, etc. The main components of this framework are the file repository, database module, resource configuration module and service module. The file repository is based on distributed Hadoop server to handle unstructured data. The authors extend Hadoop by adding the isolation of tenant’s data support and enhance its performance by improving the file repository’s ability to handle small files. The database module manages the structured data and aggregates several SQL and NoSQL database sources. The aggregation is performed through unified API and object-entity mapping for multiple databases. This considerably simplifies the development of data access modules and data exchanges for the end-user applications. The resources configuration module ensures the data management in terms of a predefined meta-model. The data resources can then be configured based on the tenant’s requirements. In addition, this module provides a set of features to manage resources usage such as data disposing and load balancing. The service module builds corresponding RESTful services from the data stored according to meta-data models and configuration patterns provided by the resources configuration module. These services are therefore used by the IoT end-user applications for their respective data management.

This work does not meet the requirement concerning the support of IoT applications heterogeneity (R1). This work does not meet the requirement concerning the support of interaction with fog layer (R2). There are no details provided about fog actuators support. This work meets the requirement concerning the federation with other IoT clouds (R3). This is done thanks to the introduced meta-models for the handled data. This work does not meet the requirement concerning the control and security support (R4). There are no details about data protection and security modules. This works does not meet the requirement concerning the support of the whole spectrum of IoT devices (R5). Only the integrated IoT devices are supported by this work since it only focus on a sub-set of functionality. Finally, this work does not meet the requirement concerning the support of virtualized IoT services (R6). It only supports the interactions with physical RFID readers and sensors.

Sii-Mobility(http://www.sii-mobility.org/) is a regional Italian city project (Tuscany region) that focus on smart mobility and transportation. In [33], the authors publish part of the project’s outcomes. The paper presents an IoT cloud architecture to enhance smart city mobility and transportation services. It enables the implementation of several scenarios that rely on IoT as big data and data analytics to operate IoT applications within a smart city setting. The designed architecture rely on four entities. The first one represents the data lake where static and real-time data are gathered. These data are acheminated to the second entity, i.e., the IoT cloud infrastructure. This entity hosts the IoT applications that are implemented as micro services. It also includes the data analytics tools that can be used through a REST API. The results can be visualized on graphical dashboards. When relevant, some (or part of) IoT applications can be deployed in the third entity of the architecture, i.e., the IoT local solution. This entity implements an edge domain, where part of the IoT applications can be placed close to the fourth entity that represents the real world with its physical IoT devices. This architecture was prototyped and several real life and complex scenario were developed and deployed over Firenze, Pisa and Prato cities to validate and evaluate this solution.

This work meets the requirement concerning the support of IoT applications heterogeneity (R1). It supports the provisioning of several smart city applications including transportation and roads signage. This work partially meets the requirement concerning the support of interaction with fog layer (R2). Although the architecture supports the provisioning of IoT applications over fog nodes, this remains limited to a subset of the connected IoT devices that are related to the architecture. The architecture does not support the discovery of other fog devices. This work meets the requirement concerning the federation with other IoT clouds (R3). On one hand, the IoT applications are interoperable since they are designed as micro services. On the other hand, the IoT devices are interoperable thanks to the IoT broker, part of the IoT cloud infrastructure entity. This work meets the requirement concerning the control and security support (R4). The architecture stands behind a firewall and authentication mechanism. Certificates and authorized credential’s keys are needed for all data sources that feed the architecture. This work meets the requirement concerning the support of the whole spectrum of IoT devices (R5) thanks to the proposed IoT description model and the IoT broker. Finally, this work does not meet the requirement concerning the support of virtualized IoT services (R6). It only supports the interactions with physical IoT devices.

## 4. PaaS for Specific IoT Verticals

**Figure 3:**
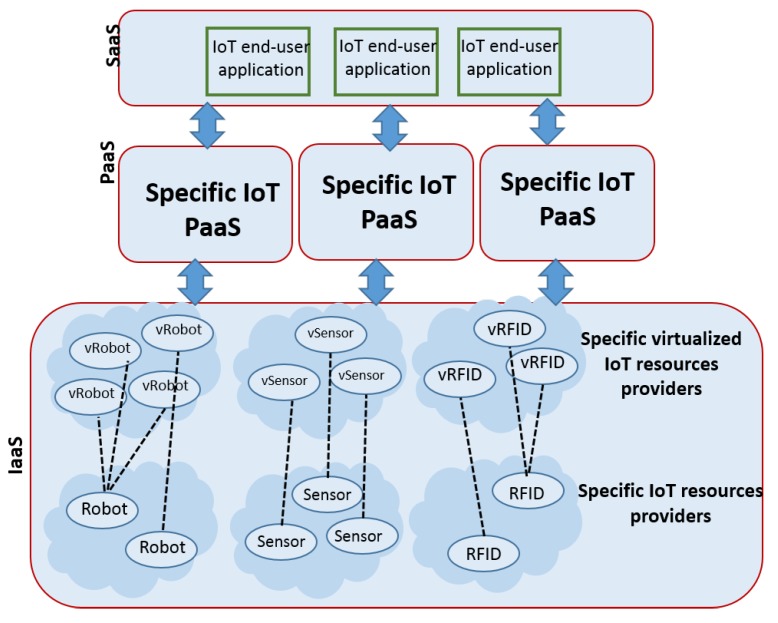
Specific IoT PaaS for specific IoT verticals.

Most of the existing IoT platforms are designed for a specific IoT verticals. As shown in Figure 3, existing solutions only provide support for domain-specific applications and/or using specific types of virtualized IoT resources. It is, therefore, obvious that these platforms do not meet the requirement concerning the support of the whole spectrum of IoT resources and technologies (R5).

The solutions in this category are organized into platforms focusing on sensing resources provisioning and those devoted to robot resources provisioning. Platforms for WSN are evaluated first. This is followed by the review of the platforms for robots.

### 4.1. PaaS for WSN

In [34], the authors propose Dinam Cloud, the PaaS for on-demand WSN end-user applications development, deployment, configuration and monitoring. The Dinam Cloud architecture consists of three layers, following the three service delivery layers of the cloud architecture: IaaS, PaaS and SaaS. The Dinam IaaS layer offers WSN computing resources (e.g., processing and storage) and is formed by heterogeneous wireless sensor nodes. The Dinam SaaS includes the concrete applications that are developed based on the Dinam PaaS to integrate WSN capabilities. In the reference implementation of the Dinam architecture, these applications are accessible via REST. The Dinam PaaS facilitates WSN end-user applications provisioning and offers unified services (e.g., storage, database and networking) to end-user applications. The Dinam PaaS reference implementation provides a programming and run-time environment for end-user applications. The programming environment is accessible via a Web-based IDE that can be used to develop new applications. The PaaS is hosted by a cloud of Dinam-mite nodes—a set of stand-alone sensor nodes that integrate the embedded Web-based development and run-time environment. Instances of the Dinam-cloud can be provisioned and released with minimal management efforts.

This work partially meets the requirement concerning the support of IoT applications heterogeneity (R1). Although the Dinam-mite concept adopted by the Dinam-cloud is very interesting, the solution discusses neither the communication interfaces among the three layers nor the interfaces for applications development and management. This work does not meet the requirement concerning the support of interaction with fog layer (R2). The Dinam-cloud architecture stack is directly running on the sensor nodes, which excludes any communications with the Dinam PaaS and the fog. This work partially meets the requirement concerning the federation with other IoT clouds (R3). It only enables federation between instances of Dinam-clouds. This work does not meet the requirement concerning the control and security support (R4). Finally, this work partially meets the requirement concerning the support of virtualized IoT services (R6). The introduced architecture shows an interaction between the PaaS and the IaaS layers that only support sensors virtualization.

Another example of PaaS for WSN is Cloud4Sens, a cloud-based platform for collecting, integrating and managing sensing capabilities from various sources [35]. These capabilities are offered to the users via two provisioning models: Data-centric and service-centric. In the data-centric model, the users are provided with access to environmental data as-a-Service. The Cloud4Sens gathers physical data and then organizes them according to an abstract and uniform format following the Sensor Web Enablement (SWE) specifications, a publicly available standard from Open Geospatial Consortium. The abstracted data is then stored and offered as-a-Service to the end-users. With the device-centric model, Cloud4Sens uses virtualization techniques to provide its users with a virtual sensing and actuation infrastructure. The users can customize the provisioned virtual devices to meet their environment monitoring needs. The bidirectional communications between the Cloud4Sens and the physical sensing infrastructure are supported via specific adapters.

This work meets the requirement concerning the support of IoT applications heterogeneity (R1). It does not meet the requirement concerning the support of interaction with fog layer (R2). Indeed, Cloud4Sens only deals with IaaS. This work meets the requirement concerning the federation with other IoT clouds (R3). Appropriate adaptors need to be added for each additional provider that will take part of the federation. This work does not meet the requirement concerning the control and security support (R4). There are no details provided neither on the IoT platform nor on the data security. Finally, it does not meet the requirement concerning the support of virtualized IoT services (R6). The Cloud4Sens architecture includes two layers, IaaS and PaaS, but these layers are used in a different perspective than in the cloud architecture. The Cloud4Sens IaaS and PaaS are independent layers used for device-centric and data-centric services, respectively. The services offered via the IaaS layer and only supports sensors virtualization.

### 4.2. PaaS for Robots

The first considered work in this category is DAvinCi [36]. It is a software framework that allows robots in large environments to collaborate in order to achieve environment exploration and map building. The sensing capabilities, such as localization and image acquisition, are distributed among the robots, which upload their sensed data to a central controller (i.e., the DAvinCi Server) to build a global map of the environment. Parts of the global map are then provided to the robots as a service on demand according to the robots needs. The computationally intense tasks of the process (here, map building) are offloaded to computing nodes in the cloud. The framework also offers a standard set of algorithms (e.g., global path planning and sensor fusion) as cloud services. These services are accessed via the DAvinCi Server, which uses the distributed Robotic Operating system (ROS) as the messaging framework to talk to the robots. ROS proxies are used to communicate with robots that cannot run ROS software.

This work does not meet the requirement concerning the support of IoT applications heterogeneity (R1). The DAvinCi framework is exclusive to map management. It also does not meet neither the requirement concerning the support of interaction with fog layer (R2) nor the requirement concerning the federation with other IoT clouds (R3) and the requirement concerning the control and security support (R4). Furthermore, the DAvinCi Server directly communicates with non-virtualized robots delivered from a specific infrastructure. Thus, it does not meet the requirement concerning the support of virtualized IoT services (R6).

In addition to DAvinCi, Rapyuta is yet another open-source robotics PaaS [37]. It enables robots to offload heavy computations to customizable and elastic computing environments in the cloud. These computing environments are tightly interconnected to allow information and service sharing among the different robots. Rapyuta’s computing environments are implemented using Linux containers that enable environments scaling up and down via the easy configuration of the computing resources assigned to each of them (e.g., memory limits). The physical robots communicate with Rapyuta using the WebSockets protocol via a full duplex and a bidirectional communications channel, which enables the platform to push information to the Robots. The exchanged messages are JSON-based. Rapytua’s internal processes intercommunicate over sockets, using the ROS inter-process communication and exchanging serialized ROS messages. Rapyuta’s processes, communicating with physical robots, convert data messages between the internal and the external communication formats (i.e., JSON and serialized ROS formats).

This work does not meet the requirement concerning the support of IoT applications heterogeneity (R1). Rapyuta focuses on offloading each robot’s computation to the cloud and then providing access to the RoboEarth database, a worldwide database in which robots can share their knowledge about objects, environments and tasks execution. It also does not meet neither the requirement concerning the support of interaction with fog layer (R2) nor the requirement concerning the federation with other IoT clouds (R3) and the requirement concerning the control and security support (R4). Finally, Rapyuta only support interactions with physical robots. Thus, it does not meet the requirement concerning the support of virtualized IoT services (R6).

Another work that could fit with this category is the framework introduced in [38] for robots virtualization. It proposes an IaaS that virtualizes physical robots and exposes them to IoT/IIoT PaaS solutions that are responsible to bind them with applications. This work presents a comprehensive business model that depicts the prospective interactions between the IaaS for robots, the PaaS solutions and their hosted applications. The considered case study corresponds to a search and rescue IoT application but the proposed architecture could support IIoT applications as well. This architecture consists of several planes. The resource plane involves the physical robots and enables the node-level virtualization. Then, it communicates with the control plane to make up the network-level robots virtualization. Basically, this level implements the services robots repository, the requests handler, the virtualization engine and all the necessary components for handling the robots-as-Service. Finally, the signaling plane contains the abstract robot gateways that could be instantiated and configured to make the bridge between robots services and the running IoT applications in the PaaS.

This work meets the requirement concerning the support of IoT applications heterogeneity (R1). The authors implemented a search and rescue application in disaster management context for validation purpose. However, the designed architecture supports any IoT application, regardless of its nature and characteristics. This work does not meet the requirement concerning the support of interaction with fog layer (R2). It only provides interfaces for IaaS/PaaS interactions. This work meets the requirement concerning the federation with other IoT clouds (R3). Other robots providers can cooperate and federate with the proposed architecture through the publication interface that enables sharing robots services and publishing them in the local robots repository. This work does not meet the requirement concerning the control and security support (R4). Finally, this work meets the requirement concerning the support of virtualized IoT services (R6) thanks to the virtual robots management interface and virtual robots operating interface it proposes.

## 5. Review Synthesis and Research Directions

This section synthesizes the evaluation results and discusses prospective research directions for the IIoT PaaS research field.

### 5.1. Evaluation Results and Main Findings

Table 1 summarizes the obtained review results in the light of the considered requirements. None of the reviewed work meets all the requirements. More specifically, not a single one of them meets neither the third requirement (i.e., interactions with IoT PaaS) nor the fourth requirement (i.e., interactions with fog layers). The research directions related to these two requirements are further discussed in this section.

### 5.2. Research Directions

This survey shows that efficient and mature IIoT PaaS solutions are still far away. Several perspectives and research directions still need to be investigated before IoT PaaS get widely accepted and used in industrial Internet. The most important research directions are discussed in the rest of this section. The selection of their related topics was motivated by the obtained evaluation results.

#### 5.2.1. Control and Security Management

Existing IIoT systems are vulnerable to a variety of cyber-attacks [39,40]. This led to make protection against cyber-attacks a major design goal for IIoT systems [41]. This concern is even more important when migrating such solutions to the cloud and provisioning them over dedicated IIoT PaaS. Indeed, achieving efficient and reliable security in IIoT PaaS is critical and mandatory to reassure and promote companies and factories to adopt and migrate their solutions to such environments. The review of the IIoT platforms highlighted that they either do not provide any security support or, when it is the case, this support is modeled as “pluggable” entity that could be transversally added to the platform like any other technical service. In addition, these “pluggable” entities often implement and adapt a classical security approach such as restoration measures of states and data (e.g., checkpointing, rollbacking and replication). However, based on [23], these approaches are too costly and complex to be implemented in IIoT systems where some devices are too restrictive in terms of computing and autonomy. This is even truer when the IIoT PaaS interacts with fog layer where devices may have the same restrictions as well. Generally speaking, there are several issues that lead to quite challenging security concerns when migrating IIoT systems to the cloud. For instance, one can cite the lack of risk mitigation, the poor management of the security updates, the use of insecure protocols and the absence of strategies following critical security incidents. The future IIoT PaaS are expected to tackle all these challenges. To that end, IIoT PaaS should adopt risk and threat management processes. These processes should be abstract and powerful enough so that they can be adapted to any industry environment. The threat model proposed in [23] can be used as starting point. In addition to that, IIoT PaaS should include asset management tools that enables identifying and discovering relevant industrial assets with regard to the deployed applications and their related SLAs/KPIs. Finally, as discussed earlier, IoT PaaS should support secure-by-design approaches that take into considerations the limitations and the specificities of the IoT devices used by the applications. Basically, a secure-by-design approach should involve the following elements: appropriate authentication method to manage identities, secure boot with the right kernel configuration and images management, secure communication using the right protocols, access control mechanism, cryptographic protection and firewall for network whitelisting.

#### 5.2.2. Interactions with Fog Layer

The integration of a fog layer would enable meeting the latency sensitivity requirement for IIoT applications when deployed in the cloud. For instance, given a distributed IIoT application, the latency-sensitive components could be provisioned over fog nodes that are close to data sources and/or actuators, and the compute-intensive components could be provisioned in the distant and more powerful IIoT PaaS [42]. This stipulates that the IIoT PaaS is able to interact with a fog layer and both make up a common and aggregated ecosystem. The literature review shows that there is still lack today of such mature system. Obviously, there are several works today that propose solutions to deploy IoT applications in hybrid cloud/fog environment (e.g., [43,44,45]). However, there are still some efforts that need to be made so that the proposed solutions fit with the CPSs at large and the IIoT in particular. A key barrier is security. Fog nodes are often owned by third parties and used by PaaS solutions in an opportunistic way. This presents a significant threat for industrial applications. The general challenges and the research directions discussed in Section 5.2.1 applies to this context as well. In addition to that and considering the hybrid cloud/fog setting, there is need to secure the control and signaling plane between these two layers. Actually, this plane is often implemented using Software-Defined Networking (SDN) entities. The security of the OpenFlow channel between the controller and its switches need to be addressed. Since all the controller commands are sent through this channel, once compromised, the network will be completely controlled by an attacker. Reference [46] describes prospective attack models in an IoT-Fog architecture. Appropriate countermeasures using Bloom filters can be considered as possible solution to this issue. When it comes to the data plane, security needs to be addressed as this level as well (e.g., encryption). furthermore, appropriate mechanisms are needed for gateways deployment and configuration. The gateways are useful to parse, convert and/or annotate the data, when needed, in between the IoT devices and the applications in hybrid cloud/fog.

Besides the security issue, another challenge is the interaction framework between the IoT PaaS in the cloud and the fog. Two research venues are worth being explored: orchestration and choreography. In the orchestration approach, the IoT PaaS could act as the orchestrator since it has an overall view. However, it is clear that this approach will not be suitable when all the IIoT application tasks are executed in the fog layer. A choreography approach would thus be better suited. It should be noted that only a few papers (e.g., [47]) acknowledge the need of orchestration between the fog and the cloud in general. However, none of them provides a solution. Finally, the reader should note that introducing the fog layer is a key enabler to achieve the tactile Internet vision [48].

#### 5.2.3. Support of Concurrent/Parallel Processes

IIoT PaaS should support various kind of processing to cover the strong heterogeneity of IIoT applications in terms of architectural design and runtime requirements. In particular, since most of the IoT applications are often made up of a set of parallel and/or concurrent processes [49], IIoT PaaS are expected to offer the appropriate tools and frameworks to support their provisioning. The reader should note that the existing IoT PaaS solutions, such as Thingworx and IBM Watson, do support concurrent/parallel processes. However, their hosting frameworks are designed and intended to end-users according to products-to-customers model where services like docker containers are offered for applications hosting and Apache Kafka for the messaging. Consequently, they are not really appropriate to IIoT setting that relies more on facilities instead of services and aim to achieve internal optimization rather than commercial products. Indeed, novel and appropriate containers need to be designed and integrated to IIoT PaaS. These containers should not only support the execution of such processes and manage the inter-communications between them (e.g., common but distributed execution context for parallel process, simultaneous access to the same shared resources for concurrent processes), but they should also implement the data and control internal workflows of the industry/factory [50]. This requires major transformation to the service containers, as well as to the communication bindings between them. The exeCution engIne for distributEd data-fLow computing (CIEL(http://www.cl.cam.ac.uk/netos/ciel/)) framework could be used as a starting point. CIEL introduces an execution engine for distributed concurrent and parallel processes [51]. However, the supported services are specific to data flow management. This framework could be extended in order to support IIoT processes. Another alternative is to extend the service micro-containers presented in reference [52]. These micro-containers were originally designed for service-based applications provisioning in the cloud. Appropriate modifications and extensions could be performed to add support of inter-processes context management.

#### 5.2.4. Interactions with IoT IaaS

Virtualization enables efficiency in resource usage. It is the key technology used today by IaaS. It is embodied in the VM concept. PaaS–IaaS interactions rely today on that concept and are enabled by classical frameworks such as OpenStack. This raises several issues. A current issue is the need to investigate the key differences between VMs and virtual IoT resources/networks. As today’s PaaS interactions with IaaS rely on the concept of VM as keystone, the more important these differences are, the more impact they will have on the IoT PaaS/IaaS interaction framework. The work introduced in [25] could be used as a starting point for the WSN vertical. It does indeed show that the differences are fundamental. These differences include the logical representation of VM vs. virtual sensors, the addressing mechanisms (IP vs. no standard mechanism) and power supply (unrestricted vs. battery operator). Similar research efforts are required for the other IoT verticals such as robots. Novel interaction frameworks are required to cater to the specifics of IoT IaaS including the differences between VMs and virtualized IoT resources/networks. They need to be investigated. Notably, such frameworks should make it possible for the IoT PaaS to interact with the IaaS at different levels of abstraction. The work in [53] might be used as a starting point to identify these different levels of abstractions. It proposes a general layering of IaaSs, made up of the following layers: cloud management, virtual infrastructure management and a VM manager [53]. Virtual IoT resources manager will of course replace the VMs manager in this case in order to support the differences that have been identified between the virtual IoT resources and VMs.

## 6. Conclusions and Perspectives

This manuscript surveys the literature on PaaS architectures for industrial Internet. It relies on a set of IIoT use cases and PaaS reference architecture to derive the evaluation criteria. The relevant papers in the literature are reviewed in the light of these criteria. Although some of these papers focus on the whole spectrum of IoT verticals, others focus on specific IoT verticals or specific IoT technologies. The architectures that target the whole spectrum of IoT verticals follow a virtual vertical-based approach, a services compositions approach, or they sometimes focus on a sub-set of functionalities. WSN and robots are the specific verticals that have been targeted so far by the proposed IIoT PaaS. None of the reviewed architectures meets all the requirements at stake. In addition to the review effort, this manuscript discusses a set of fundamental research challenges and provides hints about how they may be tackled.

In the near future, the author, with his research team, are planning to start two research projects related to this field. The first project aims at designing and prototyping an IoT hypervisor that is able to virtualize physical IoT devices and provide them as a service to IoT and IIoT cloud platforms. In a previous work [38], a specific hypervisor for robots was introduced and integrated with PaaS that provisions robots as a service. The next step is to generalize this solution and propose an IoT hypervisor that is able to virtualize the whole spectrum of IoT devices, independently of their specificities and characteristics. The second project aims at integrating Site Reliability Engineering (SRE) in IIoT cloud ecosystem. SRE relies on the software engineering concepts and applies them to infrastructure and operations problems. The ultimate goal is to design and achieve scalable and reliable systems. Integrating SRE capabilities to IIoT cloud ecosystem would considerably reduce the downtime and improve the the operational efficiency. 

## Figures and Tables

**Table 1:** Evaluated work synthesis.

References	R1	R2	R3	R4	R5	R6
[16,17]	YES	NO	Partially	YES	YES	NO
[26]	NO	Partially	NO	YES	NO	NO
[27]	YES	NO	NO	YES	YES	NO
[28]	YES	NO	NO	NO	YES	YES
[29]	YES	Partially	NO	YES	YES	NO
[30]	NO	NO	YES	NO	NO	NO
[31]	NO	NO	NO	NO	NO	NO
[32]	NO	YES	YES	NO	NO	NO
[33]	YES	Partially	YES	YES	YES	NO
[34]	Partially	NO	Partially	NO	NO	Partially
[35]	YES	NO	YES	NO	NO	Partially
[36]	NO	NO	NO	NO	NO	NO
[37]	NO	NO	NO	NO	NO	NO
[38]	YES	NO	YES	NO	YES	YES
